# The dynamics of grooming interactions: maintenance of partner choice and the consequences of demographic variation for female mandrills

**DOI:** 10.7717/peerj.6332

**Published:** 2019-01-25

**Authors:** André S. Pereira, Inês D. Rebelo, Catarina Casanova, Phyllis C. Lee, Vasilis Louca

**Affiliations:** 1 School of Biological Sciences, University of Aberdeen, Aberdeen, United Kingdom; 2 CAPP, Centro de Administração e Políticas Públicas, School of Social and Political Sciences, Universidade de Lisboa, Lisbon, Portugal; 3 CIAS, Centro de Investigação em Antropologia e Saúde, Universidade de Coimbra, Coimbra, Portugal; 4 Faculty of Natural Sciences, University of Stirling, Stirling, United Kingdom

**Keywords:** Mandrills (*Mandrillus sphinx*), Social network analysis, Grooming partner choice, Grooming reciprocity, Female grooming interactions

## Abstract

A large body of evidence suggests that female Old World monkeys maintain selective long-term grooming interactions with fitness benefits. The last two decades have produced evidence that the regulation of social interactions among primates can be, in part, explained by the Biological Markets theory, with grooming behaviour as the focus of these studies. Grooming facilitates bonding between individuals, constituting an essential part of the regulation of social relationships among female cercopithecids. In contrast to the well-studied baboons (*Papio* spp), knowledge about the nature of grooming interactions and their regulation is generally lacking for the large, terrestrial species of mandrills (*Mandrillus sphinx*). We used a combination of social network analysis tools and well-established methods for assessing partner diversity and reciprocity to characterise grooming networks, partner choice and patterns of trade (be groomed, give grooming) among females in a captive group of mandrills, both within and across two separate observation periods. Our results suggest that, even though the relatively stable conditions of captivity allowed the studied females to maintain selective grooming interactions across time, small scale demographic changes affected the grooming dynamics of the group in accordance with the expectations of the Biological Markets theory. In particular, the maturation and consequent integration of a high ranking female into the group’s grooming network from one period to the next resulted in a more pronounced effect of rank on the regulation of grooming interactions. In addition, the influence of the maturation of a dependent infant on the grooming interactions of his mother were evident between periods. Our results also demonstrate that grooming networks are dynamic and that high ranking individuals are not necessarily the most central in grooming networks. Finally, we discuss the potential of social network analysis to identify cases of social exclusion and its consequences for captive management.

## Introduction

In female kin-bonded Old World monkey species, the fitness of females has been shown to be positively affected by the formation and maintenance of strong, equitable and stable social bonds (Silk et al., 2010) with a few other specific females (Crockford et al., 2008; Silk et al., 2010; Brent et al., 2011). For female chacma baboons (*Papio ursinus*, Kerr, 1792), for example, engaging in stable focused grooming networks buffered their reaction to various external stressors (Crockford et al., 2008; Wittig et al., 2008), and maintaining strong bonds with other females increased their offspring’s survivorship (Silk et al., 2009). Indeed, in many groups of Old World monkeys, females have been shown to focus on as few as one, and up to three, preferential relationships (Engh et al., 2006; Crockford et al., 2008; Silk et al., 2012), with factors such as kinship and social rank regularly influencing partner choice (Seyfarth, 1976; Range & Noë, 2002; Silk, Alberts & Altmann, 2006; Silk, Altmann & Alberts, 2006; Schülke, Wenzel & Ostner, 2013).

Currently, one of the most discussed and tested models for the regulation of primate social interactions and preferred partner choice is the Biological Markets theory (BMT; Noë & Hammerstein, 1994). The BMT proposes that social interactions are based on the outcome of the exchange of social commodities in a marketplace and are regulated according to market forces (Hammerstein & Noë, 2016). It predicts that individuals favour the most reciprocal of their social partners according to the current demand and supply of commodities (Barrett et al., 1999; reviewed in: Hammerstein & Noë, 2016), such as tolerance (Barrett, Gaynor & Henzi, 2002), access to infants (Henzi & Barrett, 2002) or food (Fruteau et al., 2009). Hence, one key point of this model regarding the establishment of cooperative partnerships is that it posits that individuals can change their social partners if cooperating with other potential partners offers more benefits (Bshary & Noë, 2003).

According to the BMT, the time-frame for partner choice depends on the commodities traded and the stability of their value (Dunayer & Berman, 2016). If the value of the traded commodity is likely to change over short-periods, so will the value of a trading partner; whereas if the value of a commodity remains relatively stable, the value of a partner is not likely to change significantly, making it possible to have long-term trading partnerships (Dunayer & Berman, 2016). One of the proposed mechanisms for the maintenance of stable long-term partnerships in a marketplace is the emotional bookkeeping hypothesis (Aureli & Schaffner, 2002; Schino & Aureli, 2010). This hypothesis proposes that traders can form long and stable partnerships where the outcome of a single interaction is relatively meaningless to the ‘attitude’ of the traders towards each other (Aureli & Schaffner, 2002; Schino & Aureli, 2010; Hammerstein & Noë, 2016). In the context of the BMT and the emotional bookkeeping hypothesis, long-term social interactions are the outcome of preferred partners being chosen based on their market value and their interactions being regulated and maintained based on the accumulation of emotional experiences from past interactions (Hammerstein & Noë, 2016).

Even though social relationships cannot be characterised by studying a single interaction type (Smith-Aguilar et al., 2018), grooming is an ideal behaviour to investigate the regulation of social interactions in primates, as it facilitates bonding between individuals (Dunbar, 2010). Because grooming has time costs on the part of the groomer (Dunbar & Sharman, 1984; Cords, 1995) and benefits for the receiver in terms of stress reduction (Keverne, Martensz & Tuite, 1989; Aureli, Preston & De Waal, 1999) and hygiene (Tanaka & Takefushi, 1993; Zamma, 2002), it has long been central to models of partner choice, exchange and reciprocity (Saunders & Hausfater, 1988; Barrett et al., 1999; Schino & Aureli, 2010; Newton-Fisher & Lee, 2011), and social structure (Kanngiesser et al., 2011; Brent et al., 2013). Among the factors affecting partner choice and the regulation of female grooming interactions, levels of reciprocity are particularly important as these correlate with the strength and durability of social bonds (Silk, Alberts & Altmann, 2004; Silk, Alberts & Altmann, 2006; Silk, Altmann & Alberts, 2006).

The grooming-based BMT (hereafter grooming trade model: Newton-Fisher & Lee, 2011) proposes that, in social contexts where the resources can be monopolised, access to goods is mostly contingent on rank, with high ranking individuals having more access to goods than do low ranking individuals; and that grooming can be traded for itself and for other rank-related commodities, with low ranking individuals directing grooming up the hierarchy in exchange for rank-contingent goods, including social tolerance (Barrett et al., 1999; Schino, 2001; Schino, 2007; Casanova, 2002). It also predicts that in societies where the resources are equally available to all individuals, the rank of the individuals will have a weaker influence on the regulation of its trade, and grooming will mostly be reciprocally traded for itself (Barrett et al., 1999). The grooming trade model has found support in baboons, where the trade of grooming by females was in accordance with the predictions of the BMT (Barrett et al., 1999; Barrett, Gaynor & Henzi, 2002; Barrett & Henzi, 2002; Henzi & Barrett, 2002). Furthermore, females were not consistent in their social partner choice across months and years (Barrett et al., 1999; Barrett & Henzi, 2002; Henzi et al., 2009). Even though studies on the grooming trade model often investigate the reciprocal trade of grooming in the short-term, there is evidence that primates are able to maintain long-term reciprocal grooming partnerships through the emotional bookkeeping mechanism (capuchin monkeys [*Cebus apella*, Linnaeus, 1758]: Schino, Di Giuseppe & Visalberghi, 2009; mandrills [*Mandrillus sphinx*, Linnaeus, 1758]: Schino & Pellegrini, 2009).

Even though Old World monkeys, and especially baboons, are one of the most well studied groups of primates in terms of grooming (Saunders & Hausfater, 1988; Barrett et al., 1999; Zamma, 2002; Schino, Ventura & Troisi, 2003), our knowledge of females’ grooming interactions, their fitness consequences and how they are affected by factors such as dominance and kinship is still limited for wild or captive mandrills. Mandrill groups represent an opportunity to test grooming models for female Old World monkeys, as they are organised in kin-based matrilines, female social dynamics are complex and these can potentially encompass hundreds of social partners (Abernethy, White & Wickings, 2002). Within groups, females may form stable linear dominance hierarchies where younger daughters rank above their older sisters and immediately below their mother (Setchell, 2016).

Our current knowledge of grooming interactions among female mandrills suggests that captive female mandrills maintain stable long-term grooming interactions regulated by the emotional bookkeeping mechanism (Schino & Pellegrini, 2009). Schino & Lasio (2018) found competition in access to preferred grooming partners, with female mandrills preferentially directing grooming to high ranking individuals. Our understanding of social dynamics and centrality in matrilines of mandrills comes from a study of their social networks in a semi-free-ranging group of mandrills (Bret et al., 2013). Social Network Analysis (SNA) is a useful way to describe and quantify social interactions at the individual and group level (Whitehead, 2008; Sueur et al., 2011), and Bret et al. (2013) used contact as a measure of association to construct the group’s social network. They found that mandrills have preferred associates and that females associated more frequently with related individuals than unrelated individuals, whereas age and dominance rank did not influence partner choice (Bret et al., 2013). Bret et al. (2013) also found that the highest ranking females played a central role in the group’s structure, as the two females with the highest centrality scores were the highest ranking females and were of high importance to network stability and cohesion.

Because social interactions, and grooming in particular, play such an important part in the regulation of group life and in an individual’s fitness, here we investigate the grooming interactions of the females in a matrilineal group of captive mandrills. We investigate these within and across two observation periods, where one female reached sexual maturity and one infant transitioned to independence from his mother. The characteristics of the group and the timescale of the study provide an opportunity to characterise the social dynamics of a matriline of mandrills and to test the predictions of the grooming trade model. The organisation of the group into a single matriline constitutes an opportunity to investigate grooming interactions in a group whose structure mimics that of wild mandrills on a small scale, for which data are scarce and difficult to obtain (reviewed in Setchell, 2016). The captive context provides information on individual choice mechanisms given that partner choice is limited both instantaneously and over time with no migration and fewer external stressors (e.g., food limitations or predation). Finally, the chosen time-frame allows us to investigate the effect of typical demographic changes between observation periods in the group’s grooming network and individual partnerships, and to test the predictions of the grooming trade model before and after those changes. Additionally, major demographic changes that have the potential to significantly impact the group’s grooming network—such as instability in hierarchies (Dunayer & Berman, 2016; Koyama, Ronkainen & Aureli, 2017)—were unlikely to occur between periods due to the controlled environment.

Using an integrated mixed approach of SNA and conventional statistical methods, we specifically aim to understand whether the grooming interactions of the females in our study group are regulated in accordance to the predictions of the grooming trade model. We also aim to characterise three important components of grooming interactions within and across observation periods: the group’s grooming network, partner choice, and the trade of grooming for itself or other commodities in a marketplace. In order to do so, we test a set of predictions resulting from current knowledge on the regulation of grooming interactions in female Old World primates and the grooming trade model.

First, we use SNA to characterise the group’s grooming network within each period in order to understand how and if the grooming dynamics of the group changed from one period to the other in the light of demographic changes. Based on the results of the social network analysis conducted by Bret et al. (2013), we specifically predict that **(1) the highest ranking females are the most central in the grooming network**.

We predict that **(2) females engage in grooming interactions with only a few preferred partners, and partner choice is significantly maintained across observation periods**, in common with prior studies on cercopithecoids (Silk et al., 2010; Silk et al., 2012; Brent et al., 2011). The controlled captivity context of the studied group allows us to predict that, despite any possible impact of demographic changes, partner choice will be maintained due to its benefits to both partners.

We predict that, even if grooming is traded for other commodities, it constitutes a valuable commodity on its own for captive female mandrills, and thus that, overall, **(3) grooming is reciprocally traded in the studied colony**. To allow for comparability with other studies on Old World monkeys, we investigated the levels of reciprocity both within and across grooming bouts.

We also examine the effect of rank on the trade of grooming in a market place, because, when primates trade grooming for other commodities, these exchanges are monopolised by higher ranking individuals (Barrett et al., 1999). Due to the captive setting of our group, where food is abundant but available space is scarce, if grooming is traded for rank-related commodities, social tolerance (minimising the experience of aggression, supplants and avoidance) is likely to be key. Thus, we predict that, at the group level, **(4) individuals that groom each other more are more socially tolerant of each other** (i.e., are less likely to aggress, supplant and avoid one another). Again based on the premise that primates trade grooming in a market place, with higher ranking individuals receiving grooming in exchange for other rank-related commodities, we predict that **(5)** **grooming is directed up the hierarchy** and that **(6) higher ranking individuals receive more grooming than do lower ranking individuals** (e.g.,  Seyfarth, 1977; see Schino, 2001 for a meta-analysis on Ceboidea and Cercopithecoidea; chimpanzees [*Pan troglodytes*, Oken, 1816]: Kaburu & Newton-Fisher, 2015; mandrills: Schino & Lasio, 2018). Additionally, if access to rank-related benefits is rank dependent, then individuals closer in the hierarchy should have relatively similar commodities to offer each other (Barrett et al., 1999). Thus, we predict that **(7) grooming is more reciprocally traded by individuals that occupy closer positions in the hierarchy than by individuals whose rank is further apart**.

## Materials and Methods

### Study site and subjects

Data were collected in a zoological park whose non-human primates are fully habituated to the presence of human individuals. Permission to conduct research was provided by the park’s Animal Department and, because data collection consisted only of observations of the colony with no direct contact or interventions with individuals, no further ethics approval was required. All procedures in this study were performed in accordance with the European law on humane care and use of laboratory animals and the ASAB guidelines for the observation of animals (ASAB, 2018).

**Figure 1:**
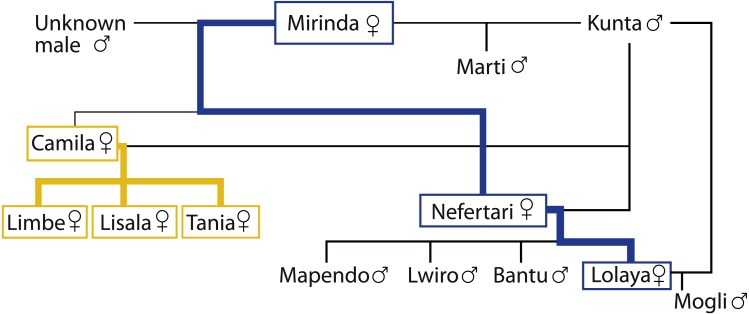
Genealogical tree of the group. The high ranking branch is represented in yellow and the low ranking branch is represented in blue.

The studied colony of mandrills was housed in the Badoca Safari Park, Herdade do Badoca, Setúbal, Portugal (38°02′26.5′N8°44′35.8′W) and consisted on 12–13 individuals. The sex, age, reproductive status and birthplace of all members of the colony were known (see Table 1 for the age and birthplace of the females), as well as their coefficient of relatedness (Fig. 1). The 6–7 mature females of the colony were the focus of the study and formed a single kin matriline (Fig. 1). At the time of both data collection periods, all focal females were under contraception by a gonadotrophin-releasing hormone agonist, which is believed to have no direct effect on the social behaviour of female Old World monkeys (EGZAC, 2014). Nevertheless, the implant minimised the influence of cycling on the social interactions of the females. Before *period one*, Nefertari had given birth to a male infant, who was still dependent on her for milk and transport during *period one*. Between periods, the number of mature females increased due to the maturation of Tania, and the total number of individuals of the colony changed due to the birth of Mogli. Lolaya gave birth to Mogli more than 15 weeks after we finished collecting data in *period one*, indicating that she was pregnant during *period one* in spite of being under contraception.

**Table 1:** Age at the beginning of data collection and birth place of the female mandrills of the studied group.

Individual	Age at period one (years)	Age at period two (years)	Birth place
Mirinda	20.9	22.6	Zoologicka Zaharada Usti na Labem (Czech Republic)
Nefertari	15.7	17.4	Barcelona Zoo (Spain)
Camila	13.9	15.6	Barcelona Zoo (Spain)
Limbe	5.2	6.8	Barcelona Zoo (Spain)
Lisala	3.7	5.3	Barcelona Zoo (Spain)
Lolaya	3.6	5.3	Barcelona Zoo (Spain)
Tania	2.3 (not observed)	4.1	Barcelona Zoo (Spain)

Zookeepers distributed vegetables, fruit and seeds in the outdoor installations before letting the group out in the morning, and a mixture of vegetables, fruit and monkey chow in the indoors installation before the mandrills returned inside in the afternoon. Water was available *ad-libitum*. Data were collected only in the outdoor enclosure, where the colony had approximately 1,674 m^2^ of available space with various enrichment devices. The indoor enclosure had an approximate size of 75 m^2^.

### Data collection

A total of 96 h of observations were collected on the colony of mandrills between the 26th of May and the 1st of July of 2016 (period one), and a further 73.5 h were collected between the 26th of January and the 8th of February of 2018 (period two). Because the subjects of the study were in a controlled environment, we expected no seasonal difference in behaviour. Data were collected through individual focal samples (Altmann, 1974; Martin & Bateson, 2007) with 16 h of focal sample on each animal in *period one*, and 10.5 h in *period two*. Each sample was of 15 min duration, and the timing and duration of all behaviours by the focal individual were recorded. When interactions between the focal individual and other individual(s) occurred, the identity of the actor(s) and recipient(s) of the interactions(s), as well as its duration, were recorded. Observations were equally distributed across all individuals. The order of observation of focal individuals was randomly decided for each day of data collection to avoid time-specific skews in observations. 10 × 20 binoculars were used.

For each dataset, the focal females of the group were organised in a linear dominance hierarchy based on the outcome (wins/losses) of dyadic supplant and avoidance interactions, supplemented with a small number of aggressive and submissive interactions. Approach-retreat interactions allow for unambiguous observation of dominance display over the supplanted individual, which is not always the case when aggression results in redirection or coalitions (Hinde, 1978). The dominance hierarchy for *period one* was based on 386 observed instances of supplant and avoidance and 33 instances of aggression/submission, whereas the hierarchy for *period two* was based on 619 supplants and avoidances with the addition of 43 aggression/submission interactions. The colony’s hierarchy was determined by calculating each individual’s average dominance index (Hemelrijk, Gygax & Wantia, 2005) and by ordering them from highest ranking to lowest ranking. Kinship was expressed by coefficient of relatedness of 0.5 between mother × daughter and sister × sister dyads, 0.375 between cousin-sibling × cousin-sibling dyads, and of 0.25 between grandmother × granddaughter dyads and aunt × niece dyads.

### Grooming network

In order to characterise the grooming network and structure of the group, we first conducted descriptive analyses. For each period we organised the total duration that each individual spent grooming another (a measure of grooming effort; Newton-Fisher & Lee, 2011) in a non-symmetric square matrix and used it to construct a directed weighted grooming network. Using Ucinet 6 (Borgatti, Everett & Freeman, 2002) we plotted a directed network diagram for each period.

Following Kanngiesser et al. (2011) and Funkhouser, Mayhew & Mulcahy (2018), we then transformed our directed grooming matrices in symmetric matrices. We did so by summing the duration of grooming traded by each dyad (i.e., the time individual A groomed individual B and the time individual B groomed individual A; Kanngiesser et al., 2011), a procedure that was justified by grooming duration being reciprocally traded at the group-level in both periods (see below; Kanngiesser et al., 2011). We then used these matrices to test for significant subgroupings within the population through community division by modularity analyses using SOCPROG 2.8 (Whitehead, 2009), with population modularity values greater than 0.30 indicating significant community structure (Newman, 2004). Posteriorly, we constructed a dendrogram for each period through hierarchical cluster analysis in SOCPROG 2.8, which was accompanied by a cophenetic correlation coefficient (CCC). CCCs greater than 0.80 indicate an appropriate visual representation of the index (Bridge, 1993). To further explore group structure, we constructed a binary matrix where dyads belonging to the same cluster were attributed a “1” and dyads belonging to different clusters were attributed a “0” (Bret et al., 2013), and correlated the matrix with the coefficient of relatedness and rank distance matrices using the tau *Kr* correlation test (Hemelrijk, 1990).

In order to test if the highest ranking females were the most central in the group’s grooming network (**prediction 1**), we used the symmetric matrices to calculate centrality measures for each individual, including: *degree* (the number of partnerships of each individual); *reach centrality* (the proportion of other individuals one individual can reach depending on the number of necessary path steps); *eigenvector centrality* (a measure of how well-connected and influential an individual is, depending both on the number and strength of its partnerships and on the influence of her associates), and *betweenness* (betweenness of an individual is defined by how many pairs can be formed using this individual as a bridge between two other individuals). These centrality measures allow us to evaluate the integration (measured by degree and reach centrality), social centrality/ power (measured by eigenvector centrality) and connectivity (measured by betweenness) of each female in the grooming network (Jacobs & Petit, 2011). We then used Spearman rank correlation tests to assess the influence of position in the dominance rank in the centrality measures. Because we are testing one hypothesis with four tests (one for each centrality measure), we corrected the significance value of each of the tests to 0.0125. For this set of tests, ranking positions were inverted (i.e., the lowest ranking female was attributed the rank of one and the highest ranking female was attributed the rank of *n*, where *n* = the number of focal females) so that significant positive relationships indicate that higher ranking individuals are more central and vice-versa. Finally, as a descriptive test to investigate how individual levels of centrality varied across periods, we used Spearman rank correlation tests to correlate the centrality values of all females present in both periods. We calculated all centrality measures with Ucinet 6 (Borgatti, Everett & Freeman, 2002).

### Grooming partner choice

In order to test if the studied females engaged in grooming interactions with only a few partners (**prediction 2**), we calculated the diversity of grooming partners for each female of the colony within both *period one* and *two* through the standardised Shannon–Weaver index (Henzi, Lycett & Weingrill, 1997; Silk, Cheney & Seyfarth, 1999; Newton-Fisher & Lee, 2011; Kaburu & Newton-Fisher, 2015):

\begin{eqnarray*}& & {H}^{{^{\prime}}}=-({p}_{i}\times \mathrm{ln} {p}_{i}+{p}_{i+1}\times \mathrm{ln} {p}_{i+1}+{p}_{i+2}\times \mathrm{ln} {p}_{i+2}+\ldots +{p}_{n}\times \mathrm{ln} {p}_{n})/(\mathrm{ln} n-1). \end{eqnarray*}

In this case, *n* is the number of mature females of the colony and *p* is the proportion of grooming frequency directed towards each female. For each female, *H*′ can vary from 0, when all grooming is focused on only one partner, to 1, when grooming is equally distributed across all females. Assessing the diversity of grooming partners describes individual variation in partner focus when the number of available partners is the same for all individuals (Newton-Fisher & Lee, 2011).

To test if partner choice was maintained across periods (**prediction 2**), we constructed a directed binary matrix for each period where, for each female (i.e., for each row), “1” corresponded to all females groomed by her and “0” to all other females; and correlated them with each other through the tau *K*_*r*_ matrix correlation test (Hemelrijk, 1990). Because Tania was not observed in *period one*, we excluded her from *period two*’s binary matrix before we correlated it with *period one*’s binary matrix. We did this so that we were able to understand if pre-existing partnerships were maintained across periods and thus, we included only the females present in both datasets in the matrices (i.e., Camila, Lisala, Limbe, Lolaya, Mirinda and Nefertari).

### Trade of grooming for itself and for other commodities

A grooming bout was defined as an interaction between two individuals where either one of them or both engaged in a continuous period of social grooming with the other (Barrett et al., 1999; Manson et al., 2004; Newton-Fisher & Lee, 2011). We considered that a grooming bout ended when more than 30 s had passed since the last social grooming between both bout participants (Newton-Fisher & Lee, 2011; Kaburu & Newton-Fisher, 2013; Kaburu & Newton-Fisher, 2015). We used either grooming duration (i.e., duration of grooming behaviour as a measure of grooming effort) or grooming frequency (i.e., participation in grooming bouts), or both in analyses. Instances of mutual grooming were considered as two separate grooming events (i.e., individual A grooms B and individual B grooms A (Lehmann & Boesch, 2009; Koyama, Ronkainen & Aureli, 2017)).

#### Grooming reciprocity

To test if grooming was reciprocally traded within bouts (**prediction 3**), we selected all grooming bouts where both members of the dyad groomed each other (i.e., all grooming bouts where grooming was immediately reciprocated). We classified each participant in a grooming dyad as Groomer (the first individual to groom) and Reciprocator (the one who reciprocates grooming) and regressed the log-transformed duration of grooming directed from the Reciprocator towards the Groomer on the log-transformed duration of grooming directed by the Groomer to the Reciprocator (Newton-Fisher & Lee, 2011). To use all available data while ensuring no dyad contributed disproportionately, we weighted the contribution each bout made to the regression by weighting each bout with the inverse function of the number of bouts by that dyad in which grooming was immediately reciprocated (following Manson et al., 2004; Newton-Fisher & Lee, 2011). To look for evidence of reciprocity across bouts when immediate reciprocity is excluded (i.e., when all grooming that was reciprocated within 30 s is excluded from the totality of traded grooming; **prediction 3**), we used the tau *K*_*r*_ test to correlate matrices of grooming given and received (Hemelrijk, 1990). To avoid missing cases where episodes of immediately reciprocated grooming contribute to across bout reciprocity, we submitted the full dataset (immediately reciprocated grooming + grooming traded across bouts) to the same tests as the data relative to grooming traded across bouts. As this replicated prior tests, we corrected the expected significance value to 0.025.

#### Tolerance

In order to investigate the relationship between grooming and social tolerance (**prediction 4**), we tested for a correlation between the directed grooming matrix and an aggression, supplant and avoidance matrix using the tau *K*_*r*_ test (Hemelrijk, 1990). We did this for both periods separately.

#### Social rank

In order to test if grooming was directed up the hierarchy (**prediction 5**), we calculated both the expected and the observed proportion of grooming that each female directed to higher ranking females and investigated whether there was a significant difference between these values for both grooming frequency and duration (based on Newton-Fisher & Lee, 2011; Kaburu & Newton-Fisher, 2013). For the expected proportion of grooming directed up the hierarchy, we calculated the total amount of grooming performed by each female and multiplied it by the proportion of females above her in the dominance hierarchy (based on Newton-Fisher & Lee, 2011; Kaburu & Newton-Fisher, 2015). We then subtracted the expected values from the observed values for each female and tested whether the difference between both values was significantly different from zero through Wilcoxon signed-ranks tests. To investigate whether higher ranking individuals received more grooming than lower ranking individuals (**prediction** **6**) we divided the amount of grooming that each female received by the amount of grooming she gave (GR/GG; in order to avoid division by zero) and compared these using Spearman’s rank correlation against female dominance rank (Kaburu & Newton-Fisher, 2015). To test if dyads closer in the hierarchy were more reciprocal (**prediction 7**), we calculated an index of reciprocity for each dyad (Newton-Fisher & Lee, 2011; Kaburu & Newton-Fisher, 2015). We adopted Mitani’s (2009) reciprocal grooming index, a rescaled version of Nishida’s (1988) index, which we labelled gRI (following Newton-Fisher & Lee, 2011):

\begin{eqnarray*}& & \mathrm{gRI}=1-{|}\mathrm{gAB}/(\mathrm{gAB}+\mathrm{gBA})-\mathrm{gBA}/(\mathrm{gAB}+\mathrm{gBA}){|}. \end{eqnarray*}

Where gAB is the time in seconds that individual A spent grooming individual B and gBA is the time in seconds that B spent grooming A. gAB + gBA is the total time, in seconds, the grooming dyad interacted. The gRI varies between 1 (perfectly reciprocal partnership) and 0 (unidirectional partnership). We then constructed a symmetric matrix with each dyad’s value of the gRI and used the tau *K*_*r*_ test (Hemelrijk, 1990) to correlate it with a matrix of each dyad’s social rank distance to test if higher values of gRI correlate with lower rank distances.

Matrix permutation tests and the average dominance indices were calculated using the MatrixTester add-in for Microsoft Excel 16 (Microsoft, Redmond, WA, USA). All other statistical analyses were conducted using Minitab 18 (Minitab Inc., State College PA, USA). All reported probabilities are two-tailed.

## Results

### Descriptive results

In *period one*, we observed grooming between 7 out of 15 possible dyads (46.67%) with one unidirectional partnership, in a total of 12,715 s of grooming distributed across 225 grooming bouts. In *period two*, we observed grooming between 9 out of 21 possible dyads (42.86%) with three unidirectional partnerships, in a total of 9,202 s of grooming distributed across 256 grooming bouts. In *period two*, Nefertari, the lowest ranking female, was not observed grooming or being groomed by any female. She died 10 days after we finished collecting data of a cardiopulmonary arrest and her autopsy found no indication of infection.

The dominance hierarchy changed between periods (Table 2). In period 2, Tania occupied second place in the rank order and Mirinda became dominant over Lolaya. In both periods, Camila and her daughters were at the top of the hierarchy, hereafter referred to as the high ranking branch; at the bottom was Mirinda, her daughter Nefertari, and Nefertari’s daughter, Lolaya, hereafter referred to as the low ranking branch. Each dyad’s gRI can be found in Table 3.

**Table 2:** Average dominance index and rank position of the focal females of the studied group.

	Period one		Period two	
Individual	Average dominance index	Rank position	Average dominance index	Rank position
Camila	1.00	1	1.00	1
Tania	–	–	0.83	2
Lisala	0.80	2	0.67	3
Limbe	0.59	3	0.50	4
Lolaya	0.41	4	0.17	6
Mirinda	0.20	5	0.33	5
Nefertari	0.00	6	0.00	7

**Table 3:** gRI values for all observed dyads.

Period one		Period two	
Dyad (rank)	gRI	Dyad (rank)	gRI
Camila (1) ×**Lisala** (2)	0.987	**Camila** (1) × Lisala (3)	0.556
Camila (1) ×**Limbe** (3)	0.975	**Camila** (1) × Limbe (4)	0.106
**Lisala** (2) × Limbe (3)	0.752	**Lisala** (3) × Limbe (4)	0.000
**Lisala** (2) × Lolaya (4)	0.147	**Lisala** (3) × Lolaya (6)	0.376
Lolaya (4) ×**Mirinda** (5)	0.779	–	
Lolaya (4) ×**Nefertari** (6)	0.000	–	
Mirinda (5) ×**Nefertari** (6)	0.147	–	
–		Camila (1) ×**Tania** (2)	0.340
–		**Camila** (1) × Mirinda (5)	0.000
–		**Tania** (2) × Lisala (3)	0.073
–		**Tania** (2) × Limbe (4)	0.000
–		**Tania** (2) × Lolaya (6)	0.099

**Notes.**

Individuals in bold received more grooming.

### Grooming network

**Figure 2:**
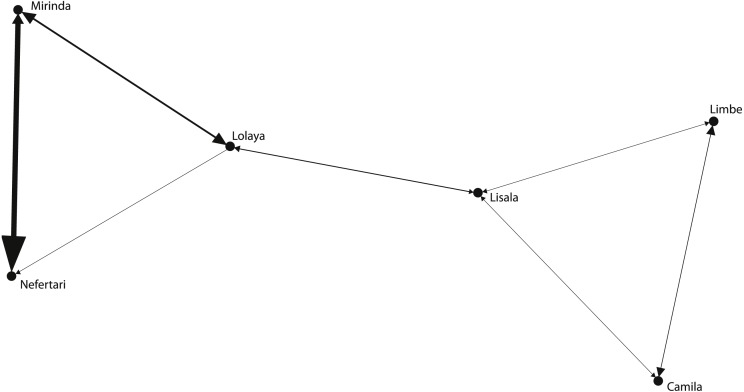
Graphic representation of the grooming network in period one. Tie width is defined by tie strength.

**Figure 3:**
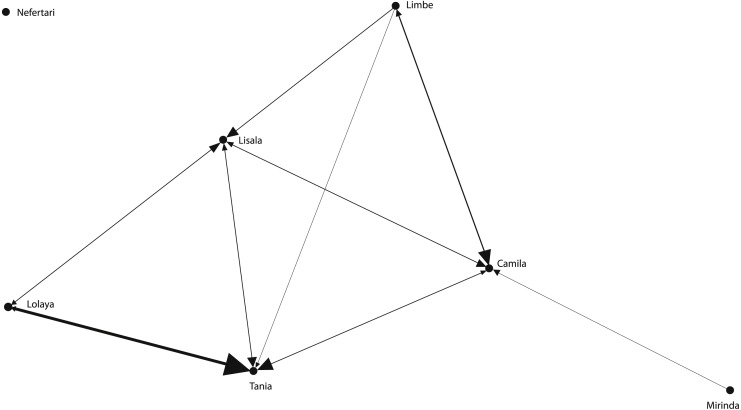
Graphic representation of the grooming network in period two. Tie width is defined by tie strength.

**Figure 4:**
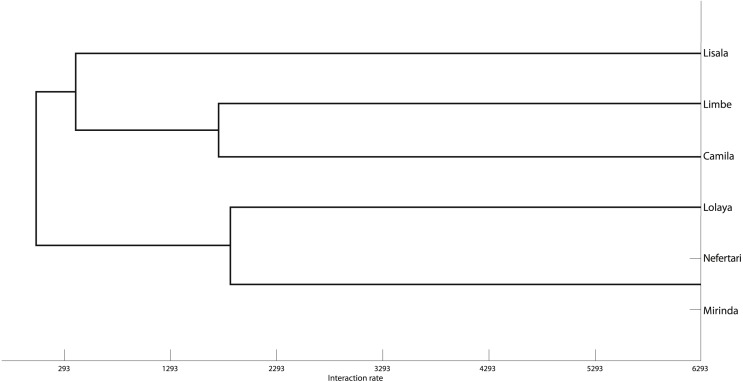
Graphic representation of the Hierarchical Cluster analysis from SNA in period one. One of the identified clusters is constituted by Lisala, Limbe and Camila, and the other by Lolaya, Nefertari and Mirinda.

**Figure 5:**
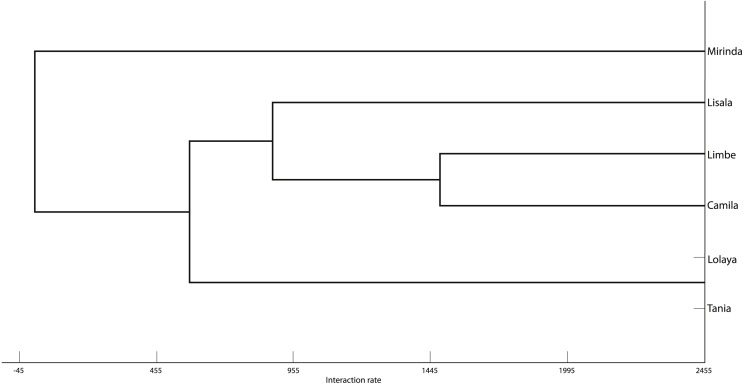
Graphic representation of the Hierarchical Cluster analysis from SNA in period two. One of the identified clusters is constituted by Mirinda, Lisala, Limbe and Camila, and the other by Lolaya and Tania.

The grooming networks for periods one and two are represented in Figs. 2 and 3, respectively. For *period one*, we identified a significant group structure with two clusters of three individuals each (maximum modularity = 0.567, CCC = 0.931; Fig. 4). These clusters were not defined by coefficient of relatedness (tau *K*_*r*_ = 0.531, *N* = 6, *p* = 0.195) or proximity in dominance rank (tau *K*_*r*_ =  − 0.636, *N* = 6, *p* = 0.102). For *period two*, we also identified two clusters, one with two individuals and the other with four (maximum modularity = 0.329, CCC = 0.871; Fig. 5). Nefertari was not attributed to any cluster because she did not engage in any grooming interaction with any group member. These clusters again were not defined by coefficient of relatedness (tau *K*_*r*_ = 0.140, *N* = 6, *p* = 0.280) or proximity in dominance rank (tau *K*_*r*_ = 0.097, *N* = 6, *p* = 0.726).

Our results provide only limited support to our **prediction 1** as, apart from a significant positive correlation between higher rank position and higher degree (*r*_S_ = 0.927, *N* = 7, *p* = 0.003), reach centrality (*r*_S_ = 0.927, *N* = 7, *p* = 0.003) and betweenness (*r*_S_ = 0.896, *N* = 7, *p* = 0.006) in *period two*, there was no correlation between rank and any other centrality measures for *period one* (*degree*: *r*_S_ = 0.207, *N* = 6, *p* = 0.694; *reach centrality*: *r*_S_ = 0.207, *N* = 6, *p* = 0.694; *eigenvector centrality*: *r*_S_ =  − 0.771, *N* = 6, *p* = 0.072; *betweenness*: *r*_S_ = 0.207, *N* = 6, *p* = 0.694) or *period two* (*eigenvector centrality*: *r*_S_ = 0.571, *N* = 7, *p* = 0.180). Individual centrality measures (Table 4) for *period one* were not correlated with the measures obtained for *period two* (*degree*: *r*_S_ = 0.315, *N* = 6, *p* = 0.543; *reach centrality*: *r*_S_ = 0.315, *N* = 6, *p* = 0.543; *eigenvector centrality*: *r*_S_ =  − 0.429, *N* = 6, *p* = 0.397; *betweenness*: *r*_S_ = 0.122, *N* = 6, *p* = 0.817).

**Table 4:** Individual centrality measures from social network analysis of grooming interactions.

Period	Individual	Degree	Reach centrality	Eigenvector centrality	Betweenness
One	Camila	2	4.167	0.001	0.000
	Lisala	3	5.000	0.009	6.000
	Limbe	2	4.167	0.000	0.000
	Lolaya	3	5.000	0.356	6.000
	Mirinda	2	4.167	**0.704**	0.000
	Nefertari	2	4.167	0.614	0.000
Two	Camila	4	5.500	0.446	**4.000**
	Tania	4	5.500	**0.589**	1.500
	Lisala	4	5.500	0.404	1.500
	Limbe	3	5.000	0.296	0.000
	Mirinda	1	3.833	0.006	0.000
	Lolaya	2	4.333	0.451	0.000
	Nefertari	0	1.000	0.000	0.000

**Notes.**

Values in bold indicate the highest isolated value in each column for each measure.

### Grooming partner choice

The results provide strong support to **prediction 2**. In *period one*, the six mature females groomed only between one and three other individuals each, and grooming diversity index values were low (*H*′): mean = 0.299 ± 0.06; Table 5). In *period two*, the focal females groomed between zero and three other females and grooming diversity index values were also low (*H*′): mean = 0.317 ± 0.08; Table 5). Between periods, females significantly maintained their partner choice (tau *K*_*r*_ = 0.576, *N* = 6, *p* = 0.006).

**Table 5:** Individual grooming preferences and *H*′ in both periods.

Groomer	Period one		Period two	
	Grooming partners	*H*′	Grooming partners	*H*′
Camila	Lisala, Limbe	0.427	Tania, Lisala, Limbe	0.345
Tania	–	–	Camila, Lisala, Lolaya	0.543
Lisala	Camila, Limbe, Lolaya	0.393	Camila, Tania, Lolaya	0.498
Limbe	Camila, Lisala	0.292	Camila, Tania, Lisala	0.511
Lolaya	Lisala, Mirinda, Nefertari	0.396	Lisala, Tania	0.325
Mirinda	Lolaya, Nefertari	0.284	Camila	0.000
Nefertari	Mirinda	0.000	–	0.000

### Trade of grooming for itself and for other commodities

#### Grooming reciprocity

When all data were included in analyses, grooming was reciprocally traded in both *period one* (*effort*: tau *K*_*r*_ = 0.856, *N* = 6, *p* = 0.003; *frequency*: tau *K*_*r*_ = 0.902, *N* = 6, *p* = 0.001) and *period two* (*effort*: tau *K*_*r*_ = 0.386, *N* = 6, *p* = 0.043; *frequency*: tau *K*_*r*_ = 0.675, *N* = 6, *p* = 0.002), justifying our procedure of summing the given and received matrices for the grooming network analyses (see ‘Materials and Methods’ section). However, given repeated hypotheses, when we corrected the expected probability to 0.025, **prediction 3** was only partially supported. Within bouts, the effort put by the Groomer was not significantly reciprocated in *period one* (*F*_1,13_ = 5.52, *s*^2^ = 0.322, *p* = 0.035), but was in *period two* (*F*_1,17_ = 8.31, *s*^2^ = 0.189, *p* = 0.010). In *period one*, when bouts of immediately reciprocated grooming were excluded, there was reciprocity in grooming given and received across all grooming bouts of all dyads (*grooming effort*: tau *K*_*r*_ = 0.902, *N* = 6, *p* = 0.002; *grooming frequency*: tau *K*_*r*_ = 0.902, *N* = 6, *p* = 0.002). In *period two*, the results for grooming frequency were similar (tau *K*_*r*_ = 0.464, *N* = 6, *p* = 0.007); however, grooming effort was not reciprocally traded when we excluded all instances of immediately reciprocated grooming (tau *K*_*r*_ = 0.233, *N* = 6, *p* = 0.153).

#### Tolerance

We found a significant negative correlation between given and received grooming effort and given and received aggression, supplants and avoidance for both periods (*period one*: tau *K*_*r*_ =  − 0.209, *N* = 6, *p* = 0.020; *period two*: tau *K*_*r*_ =  − 0.531, *N* = 6, *p* = 0.005), confirming our prediction that females who groomed each other more were more tolerant of each other (**prediction 4**).

#### Social rank

In *period one*, only 25.76% of total grooming effort and 32.89% of total grooming frequency was directed up the hierarchy. These small values, however, seem to have been caused by the low ranking branch. At the time of data collection, Nefertari, the lowest ranking female, was mother to a dependent infant, and the other two low ranking females spent more time grooming her than she groomed them. Because of the potentially distorting effect of infants on their mother’s grooming interactions (Henzi & Barrett, 2002), we excluded grooming to and from Nefertari and found that 44.86% of total grooming effort and 45.26% of total grooming frequency was then directed up the hierarchy. Even though only less than half of the grooming was directed up the hierarchy, **prediction 5** was supported as there was no significant difference between the expected grooming up the hierarchy and the observed grooming up the hierarchy (i.e., the difference between the expected and observed grooming directed up the hierarchy did not differ from zero; Wilcoxon test: *effort*: *Z* = 3.00, *p* = 1.000, *frequency*: *Z* = 3.00, *p* = 1.000). In *period two*, 78.93% of total grooming effort and 60.94% of total grooming frequency was directed up the hierarchy. The difference between the observed values and the expected values was significantly greater than zero (Wilcoxon test: *effort*: *Z* = 15.00, *p* = 0.030, *frequency*: *Z* = 15.00, *p* = 0.030), and thus, significantly more grooming than expected was directed up the hierarchy.

We did not find support for **prediction 6** in *period one*, as we did not find evidence that high ranking females received more grooming than low ranking females (*r*_S_ =  − 0.029, *N* = 6, *p* = 0.957). However, social rank affected the levels of received grooming in *period two*, with higher ranking females receiving more grooming than lower ranking females (*r*_S_ = 0.829, *N* = 7, *p* = 0.021), partially confirming our prediction.

Finally, we found no support for **prediction 7** as there was no relationship between proximity of ranks in the hierarchy and level of reciprocity in *period one* (tau *K*_*r*_ =  − 0.633, *N* = 6, *p* = 0.059) or *period two* (tau *K*_*r*_ =  − 0.049, *N* = 7, *p* = 0.471).

## Discussion

Primate social networks are dynamic, and the extent to which they remain stable over time and how long it takes for them to stabilise after change remains unclear (Borgeaud et al., 2017). Due to the sexual maturation of one female and her consequent integration into the adult grooming network, as well as to the maturation of an infant from one period to the next, we expected the network of the studied group to be relatively dynamic. Demographic events such as these (Barrett & Henzi, 2002) impact the fabric of social interactions in any primate group, and the collection of focal data in two short but distinct periods allowed us to assess the dynamics and stability of the group’s grooming network while minimising the risk of social network change within each period and its confounding variation (Levé et al., 2016). Even though limited time-frames might obscure some levels of grooming reciprocity (Gomes, Mundry & Boesch, 2009), the time-frame for long-term grooming reciprocation remains poorly contextualised, and whether it varies between species or groups needs further analysis. Our results indicate that grooming was generally reciprocally traded in both periods, suggesting that a relevant time-frame for grooming reciprocation was included in the time-span of the study. We illustrate the outcome of our general predictions in Table 6. Here we will discuss when and why these predictions were confirmed or refuted.

**Table 6:** Summary of the predictions and our results.

Prediction	Findings
(1) The highest ranking females are the most central in the grooming network.	Rejected for *period one* and partially confirmed for *period two*. Degree, reach centrality and betweenness in *period two* were the only centrality measures that correlated with rank. Eigenvector centrality did not correlate with rank in *period two*; however, the female with the highest value was a high ranking individual.
(2) Females engage in grooming interactions with only a few preferred partners, and partner choice is significantly maintained across observation periods.	Confirmed. For both periods, females groomed only between 0–3 other individuals, and partner preference was significantly maintained across periods.
(3) Grooming is reciprocally traded in the studied colony.	Partially confirmed. In *period one*, overall grooming was reciprocally traded despite the lack of reciprocity within bouts. In *period two*, grooming was reciprocally traded within bouts, whereas grooming effort was overall non-reciprocally traded.
(4) Individuals that groom each other more are more socially tolerant of each other.	Confirmed. We found a significant correlation between given and received grooming and aggression, supplants and avoidance in both periods.
(5) Grooming is directed up the hierarchy.	Partially confirmed. In *period one*, grooming was directed up the hierarchy only after we removed all grooming to and from Nefertari, who had a dependent infant. Confirmed for *period two.*
(6) Higher ranking individuals receive more grooming than do lower ranking individuals.	Confirmed for *period two* but not for *period one*.
(7) Grooming is more reciprocally traded by individuals that occupy closer positions in the hierarchy than by individuals whose rank is further apart.	Rejected for both periods.

The SNA was useful to characterise the grooming network of the group within each period and to contextualise the individual partnerships at the group level. In *period one*, SNA indicated that the group was divided in two clusters, representing the low ranking branch and the high ranking branch of the matriline. Lisala and Lolaya’s partnership played a central role in connecting the network as theirs was the only partnership connecting both clusters (Fig. 2). Indeed, Lisala and Lolaya both had the highest betweenness values, and, as defined by Lusseau & Newman (2004), the individuals with highest betweenness values act as brokers that connect sub-communities and who are crucial to the cohesion of the population. On the other hand, in *period two*, we found a cluster connecting all females that engaged in grooming (Fig. 3). Contrary to findings from *period one*, in *period two*, higher ranking females were significantly more integrated and connected than were lower ranking females, and, even though rank was uncorrelated with power across all individuals (measured by eigenvector centrality), the most powerful individual was a high ranking female. Thus, regarding the effect of rank on the levels of centrality (**prediction 1**), it is clear that rank had a bigger influence in *period two* than in *period one*. Further supporting this observation, Mirinda (fifth in the rank in both observation periods), who was the most powerful individual in *period one*, was integrated in the network of *period two* only by a weak unidirectional partnership with Camila; and Nefertari (last in the rank in both periods) was totally excluded in *period two*.

SNA-based studies in mandrills are still rare (Bret et al., 2013; Naud et al., 2016) and absent in wild groups. It is likely that social networks will vary between different groups and over time due to the personality of individuals or group composition, as reported for chimpanzees (Van Leeuwen et al., 2013; Cronin et al., 2014; Levé et al., 2016). Additionally, Bret et al. (2013) measured centrality in an association network, and centrality may be a function of the nature of the interaction, with the more central individuals in terms of association being different from central interactants—either more aggressive or more affiliative (e.g., Funkhouser, Mayhew & Mulcahy, 2018; Smith-Aguilar et al., 2018). We also found that the centrality levels of each individual changed significantly from one period to the other, further evidencing how dynamic and variable social networks can be. SNA constitutes a useful way to investigate a group’s structure and the interactions between its members, and the analyses we conducted here allowed us to illustrate the studied group’s grooming interactions and the position of each female within the grooming network in each period. However, results obtained from SNA based on a single type of social interaction must be interpreted with caution as they do not represent the full complexity of female social relationships (Smith-Aguilar et al., 2018).

With respect to partner choice (**prediction 2**), despite some variation in the dyads observed across periods, females had clear preferences within each period when it came to choosing their grooming partners, and partner choice was significantly maintained across periods, supporting the proposal that grooming only some specific individuals is a common strategy across Old World monkeys (Engh et al., 2006; Crockford et al., 2008; Silk et al., 2012). Even though group life is inevitably dynamic, and demographic and stochastic factors have the potential to influence social partnerships (Barrett & Henzi, 2002), our results suggest that the relatively stable environment of the study group and the absence of major demographic shifts between the two observation periods allowed the females to maintain their previously established grooming partnerships (Dunayer & Berman, 2016; Hammerstein & Noë, 2016). The observed maturation of an infant and integration of a female into the hierarchy constitute small scale demographic changes that are normal to primates’ group life, and if social partnerships can potentially last a life-time (Hammerstein & Noë, 2016), then it is likely that normal demographic changes will not significantly affect the overall maintenance of previously established grooming partnerships within a group.

As for levels of grooming reciprocity (**prediction 3**), our results for the immediacy of reciprocation of grooming in *period one* and the overall maintenance of partner choice across periods suggest that an emotional bookkeeping mechanism might have, in fact, been regulating the grooming interactions of the studied group. Our findings for *period one* replicate those obtained by Schino & Pellegrini (2009), who found that, even though grooming was overall reciprocally traded among the females of a captive group of mandrills, its trade within bouts was not reciprocal. Schino & Pellegrini (2009) collected data over a long time-frame and were able to conclude that the outcome (i.e., the observed level of reciprocity) of immediately traded grooming was most likely meaningless to the long-term regulation of each dyad’s grooming partnership. We suggest that this was probably the case for our study group. Our findings for the reciprocity of grooming in *period two* are contrary to those of *period one*; however, it is likely that the positive emotions that contribute to the bookkeeping mechanism result from all interactions between individuals (grooming, tolerance, support in agonism, etc; Schino & Aureli, 2009), thus, it is possible that the potential negative effect of the lack of grooming reciprocity in *period two* was compensated by the trade of other rank related commodities.

The effect of rank on the trade of grooming (**predictions 5–7**) was complex; the observed lack of reciprocity in the trade of grooming time across bouts in *period two* suggests that there was a stronger effect of rank on grooming trade in this period, associated with the inclusion of Tania into the hierarchy. With unbalanced dominance relationships, grooming given by higher ranking individuals is more valuable than that given by lower ranking individuals (Barrett et al., 1999) and thus, high ranking individuals may be subject to more grooming from low ranking individuals with little need to reciprocate, as apparent in *period two.* The change in rank differentials also led to a more pronounced effect of rank on the centrality of individuals in *period two.*

Regarding the trade between grooming and social tolerance (**prediction 4**), the grooming trade model predicts that higher power differentials potentiate the trade of grooming for rank-related commodities (Barrett et al., 1999), and we found evidence that grooming may have been traded for social tolerance as assessed by the negative association between grooming and agonistic interactions in *period two*. In spite of the higher levels of reciprocity observed in *period one*, it is likely that at least some grooming was being traded for other commodities, such as tolerance. Our analysis is correlational in nature and, because grooming facilitates bonding (reviewed in Dunbar, 2010), our findings may be the result of an existing friendship rather than an actual trade between grooming and tolerance in a marketplace.

Whereas the maturation of Tania affected the grooming patterns at the group level, the maturation of Nefertari’s infant was mostly visible at the dyadic level. In *period one*, the grooming interactions of Nefertari were unbalanced (Table 3), supporting the expectations of a grooming trade model mediated by a “baby market” in which she received grooming in exchange for access to the infant (Henzi & Barrett, 2002). With the infant’s independence, however, her value as a grooming partner clearly changed, supporting Dunayer & Berman (2016) suggestion that infant access constitutes a “volatile” commodity whose value in the market can change in the short-term.

Finally, our findings also have implications for the welfare and management of captive groups of primates. SNA tools provide useful insights regarding the cohesion of captive groups and have often been used to evaluate individuals’ well-being, establishing a basis for informed decision making in the management of captive groups (McCowan et al., 2008; Funkhouser, Mayhew & Mulcahy, 2018) while also allowing formal evaluation of management decisions (Clark, 2011). Our results suggest SNA to be particularly useful in identifying and investigating cases of social exclusion. In the wild, social exclusion often comes with fitness consequences resulting in the migration of the ostracised individual and being associated with increased rates of mortality (Lancaster, 1986). In captivity, migration is not an option for socially excluded individuals, and cases of social exclusion must be promptly identified to avoid potential fitness consequences. As an example, the female Nefertari, who was excluded from the grooming network of the group in *period two* and was actively aggressively pursued by the alpha male (personal observations) and the highest ranking females (Data S1), died from no apparent cause 10 days after data collection ceased.

## Conclusions

Here, we characterised the grooming interactions of female mandrills regarding three of its main aspects: grooming network, partner choice, and the trade of grooming in a marketplace. Even though not all our predictions were fully supported, our results provide evidence of how grooming interactions are regulated among the females in a single matriline of mandrills. Overall, our results suggest that female mandrills engage in grooming interactions with only a few partners, who are mostly maintained across time when demographic conditions remain relatively stable, that those interactions can be explained by the BMT and that grooming may be traded for social tolerance. Furthermore, we characterised how the presence of a dependent infant influenced its mother’s grooming interactions, and our results suggest that the predictions of a grooming-mediated baby market may also apply to groups of mandrills. Our results also suggest that the sexual maturation of a female increased the effect of rank on the observed patterns of grooming, supporting the BMT’s prediction that a higher rank differential between top ranking and low ranking individuals leads to overall unbalanced grooming interactions. Finally, we emphasise the potential of social network analysis to investigate the welfare of captive groups of mandrills, and particularly to identify cases of social exclusion. Due to the captive setting of the studied group, the small sample size and our focus on females, our capacity to generalise the obtained results to wild groups is limited. However, given that mandrills are very difficult to follow in the wild (Setchell, 2016), that the organisation of the studied group in a matriline reflects that of wild groups (Abernethy, White & Wickings, 2002) and that our knowledge on the regulation of grooming interactions in mandrills is still limited, we believe that our findings make a valuable contribution to our understanding of mandrill grooming interactions. Furthermore, primates form particularly socially complex groups (Silk, 2007), and our results highlight just how dynamic grooming interactions can be, suggesting it may not be prudent to generalise results obtained from a single group.

##  Supplemental Information

10.7717/peerj.6332/supp-1 Data S1 Grooming, supplant and avoidance, and aggression data

## Additional Information and Declarations

### Field Study Permissions

The following information was supplied relating to field study approvals (i.e., approving body and any reference numbers): Field work was carried under the form of two data collection periods on the mandrill colony of Badoca Safari Park, Portugal, in 2016 and in 2018. Permission to collect data in 2016 was granted verbally by Alfredo Gargallo Martín, Director of the Animal Department of Badoca Safari Park at the time. In 2018, permission to collect data was granted verbally by Rui Cunha, the Technical Director of the Animal Department of Badoca Safari Park.

### Data Availability

The following information was supplied regarding data availability: The raw data are available in a Supplemental File.
