# Supplementary material for: The dynamics of grooming interactions: maintenance of partner choice and the consequences of demographic variation for female mandrills
Source: PeerJ. 2019 Jan 25;7:e6332. doi: 10.7717/peerj.6332 (PMC6348956; doi:10.7717/peerj.6332)
Supplement: Data S1 [file peerj-07-6332-s001.docx]

**Data collected on 2016**

**Grooming effort**

All traded grooming (duration in seconds)

|  | Camila | Lisala | Limbe | Lolaya | Mirinda | Nefertari |
| --- | --- | --- | --- | --- | --- | --- |
| Camila | 0 | 312 | 894 | 0 | 0 | 0 |
| Lisala | 304 | 0 | 65 | 13 | 0 | 0 |
| Limbe | 850 | 108 | 0 | 0 | 0 | 0 |
| Lolaya | 0 | 164 | 0 | 0 | 2176 | 147 |
| Mirinda | 0 | 0 | 0 | 1389 | 0 | 5832 |
| Nefertari | 0 | 0 | 0 | 0 | 461 | 0 |

Across bout traded grooming (after removing immediately traded grooming; duration in seconds)

|  | Camila | Lisala | Limbe | Lolaya | Mirinda | Nefertari |
| --- | --- | --- | --- | --- | --- | --- |
| Camila | 0 | 297 | 574 | 0 | 0 | 0 |
| Lisala | 292 | 0 | 65 | 13 | 0 | 0 |
| Limbe | 590 | 108 | 0 | 0 | 0 | 0 |
| Lolaya | 0 | 164 | 0 | 0 | 314 | 147 |
| Mirinda | 0 | 0 | 0 | 159 | 0 | 5226 |
| Nefertari | 0 | 0 | 0 | 0 | 318 | 0 |

Bouts of immediately traded grooming (within-bout grooming duration)

| Groomer | Reciprocator | Grooming given by Groomer (seconds) | Grooming reciprocated by Reciprocator (seconds) |
| --- | --- | --- | --- |
| Lolaya | Mirinda | 526 | 354 |
| Mirinda | Nefertari | 36 | 77 |
| Mirinda | Lolaya | 591 | 304 |
| Camila | Limbe | 145 | 74 |
| Camila | Lisala | 15 | 12 |
| Lolaya | Mirinda | 747 | 114 |
| Mirinda | Lolaya | 41 | 75 |
| Mirinda | Lolaya | 47 | 68 |
| Limbe | Camila | 86 | 3 |
| Mirinda | Lolaya | 83 | 142 |
| Limbe | Camila | 77 | 39 |
| Camila | Limbe | 133 | 23 |
| Mirinda | Nefertari | 287 | 36 |
| Mirinda | Nefertari | 182 | 16 |
| Mirinda | Nefertari | 101 | 14 |

**Grooming frequency**

All traded grooming (number of grooming episodes)

|  | Camila | Lisala | Limbe | Lolaya | Mirinda | Nefertari |
| --- | --- | --- | --- | --- | --- | --- |
| Camila | 0 | 19 | 24 | 0 | 0 | 0 |
| Lisala | 15 | 0 | 3 | 1 | 0 | 0 |
| Limbe | 23 | 5 | 0 | 0 | 0 | 0 |
| Lolaya | 0 | 4 | 0 | 0 | 28 | 3 |
| Mirinda | 0 | 0 | 0 | 15 | 0 | 73 |
| Nefertari | 0 | 0 | 0 | 0 | 12 | 0 |

Across bout traded grooming (after removing immediately traded grooming; number of grooming episodes)

|  | Camila | Lisala | Limbe | Lolaya | Mirinda | Nefertari |
| --- | --- | --- | --- | --- | --- | --- |
| Camila | 0 | 18 | 19 | 0 | 0 | 0 |
| Lisala | 14 | 0 | 3 | 1 | 0 | 0 |
| Limbe | 18 | 5 | 0 | 0 | 0 | 0 |
| Lolaya | 0 | 4 | 0 | 0 | 18 | 3 |
| Mirinda | 0 | 0 | 0 | 5 | 0 | 66 |
| Nefertari | 0 | 0 | 0 | 0 | 8 | 0 |

**Supplants and avoidances** (number of episodes)

|  | Camila | Lisala | Limbe | Lolaya | Mirinda | Nefertari |
| --- | --- | --- | --- | --- | --- | --- |
| Camila | 0 | 6 | 4 | 4 | 7 | 54 |
| Lisala | 0 | 0 | 32 | 20 | 15 | 76 |
| Limbe | 0 | 0 | 0 | 15 | 31 | 106 |
| Lolaya | 0 | 0 | 1 | 0 | 1 | 12 |
| Mirinda | 0 | 0 | 0 | 0 | 0 | 2 |
| Nefertari | 0 | 0 | 0 | 0 | 0 | 0 |

**Aggression** (number of episodes)

|  | Camila | Lisala | Limbe | Lolaya | Mirinda | Nefertari |
| --- | --- | --- | --- | --- | --- | --- |
| Camila | 0 | 1 | 3 | 6 | 0 | 2 |
| Lisala | 0 | 0 | 0 | 2 | 0 | 0 |
| Limbe | 0 | 0 | 0 | 12 | 1 | 6 |
| Lolaya | 0 | 0 | 0 | 0 | 0 | 0 |
| Mirinda | 0 | 0 | 0 | 0 | 0 | 0 |
| Nefertari | 0 | 0 | 0 | 0 | 0 | 0 |

**Data collected on 2018**

**Grooming effort**

All traded grooming (duration in seconds)

|  | Camila | Tania | Lisala | Limbe | Mirinda | Lolaya | Nefertari |
| --- | --- | --- | --- | --- | --- | --- | --- |
| Camila | 0 | 1293 | 243 | 79 | 0 | 0 | 0 |
| Tania | 265 | 0 | 36 | 0 | 0 | 121 | 0 |
| Lisala | 631 | 948 | 0 | 0 | 0 | 134 | 0 |
| Limbe | 1411 | 200 | 879 | 0 | 0 | 0 | 0 |
| Mirinda | 49 | 0 | 0 | 0 | 0 | 0 | 0 |
| Lolaya | 0 | 2334 | 579 | 0 | 0 | 0 | 0 |
| Nefertari | 0 | 0 | 0 | 0 | 0 | 0 | 0 |

Across bout traded grooming (after removing immediately traded grooming; duration in seconds)

|  | Camila | Tania | Lisala | Limbe | Mirinda | Lolaya | Nefertari |
| --- | --- | --- | --- | --- | --- | --- | --- |
| Camila | 0 | 1205 | 91 | 79 | 0 | 0 | 0 |
| Tania | 87 | 0 | 14 | 0 | 0 | 0 | 0 |
| Lisala | 395 | 926 | 0 | 0 | 0 | 100 | 0 |
| Limbe | 1411 | 200 | 879 | 0 | 0 | 0 | 0 |
| Mirinda | 49 | 0 | 0 | 0 | 0 | 0 | 0 |
| Lolaya | 0 | 1517 | 356 | 0 | 0 | 0 | 0 |
| Nefertari | 0 | 0 | 0 | 0 | 0 | 0 |  |

Bouts of immediately traded grooming (within-bout grooming duration)

| Groomer | Reciprocator | Grooming given by Groomer (in seconds) | Grooming reciprocated by Reciprocator (in seconds) |
| --- | --- | --- | --- |
| Lolaya | Tania | 103 | 5 |
| Lolaya | Tania | 47 | 20 |
| Tania | Lolaya | 17 | 60 |
| Tania | Camila | 85 | 12 |
| Lisala | Lolaya | 7 | 134 |
| Tania | Lolaya | 12 | 25 |
| Tania | Camila | 43 | 9 |
| Camila | Tania | 34 | 23 |
| Lisala | Lolaya | 12 | 45 |
| Tania | Lolaya | 1 | 57 |
| Lolaya | Tania | 199 | 34 |
| Lolaya | Tania | 230 | 13 |
| Lisala | Tania | 22 | 22 |
| Tania | Lolaya | 19 | 96 |
| Lolaya | Lisala | 44 | 15 |
| Lisala | Camila | 20 | 99 |
| Lisala | Camila | 130 | 37 |
| Camila | Tania | 33 | 27 |
| Lisala | Camila | 86 | 16 |

**Grooming frequency**

All traded grooming (number of grooming episodes)

|  | Camila | Tania | Lisala | Limbe | Mirinda | Lolaya | Nefertari |
| --- | --- | --- | --- | --- | --- | --- | --- |
| Camila | 0 | 66 | 13 | 4 | 0 | 0 | 0 |
| Tania | 12 | 0 | 3 | 0 | 0 | 10 | 0 |
| Lisala | 29 | 26 | 0 | 0 | 0 | 4 | 0 |
| Limbe | 11 | 2 | 12 | 0 | 0 | 0 | 0 |
| Mirinda | 1 | 0 | 0 | 0 | 0 | 0 | 0 |
| Lolaya | 0 | 46 | 17 | 0 | 0 | 0 | 0 |
| Nefertari | 0 | 0 | 0 | 0 | 0 | 0 | 0 |

Across bout traded grooming (after removing immediately traded grooming; number of grooming episodes)

|  | Camila | Tania | Lisala | Limbe | Mirinda | Lolaya | Nefertari |
| --- | --- | --- | --- | --- | --- | --- | --- |
| Camila | 0 | 62 | 9 | 4 | 0 | 0 | 0 |
| Tania | 8 | 0 | 2 | 0 | 0 | 1 | 0 |
| Lisala | 23 | 25 | 0 | 0 | 0 | 1 | 0 |
| Limbe | 11 | 2 | 12 | 0 | 0 | 0 | 0 |
| Mirinda | 1 | 0 | 0 | 0 | 0 | 0 | 0 |
| Lolaya | 0 | 34 | 13 | 0 | 0 | 0 | 0 |
| Nefertari | 0 | 0 | 0 | 0 | 0 | 0 | 0 |

**Supplants and avoidances** (number of episodes)

|  | Camila | Tania | Lisala | Limbe | Mirinda | Lolaya | Nefertari |
| --- | --- | --- | --- | --- | --- | --- | --- |
| Camila | 0 | 4 | 9 | 15 | 8 | 19 | 4 |
| Tania | 0 | 0 | 79 | 55 | 13 | 33 | 10 |
| Lisala | 0 | 1 | 0 | 109 | 23 | 23 | 7 |
| Limbe | 0 | 0 | 0 | 0 | 103 | 67 | 27 |
| Mirinda | 0 | 0 | 0 | 0 | 0 | 4 | 2 |
| Lolaya | 0 | 0 | 0 | 0 | 0 | 0 | 4 |
| Nefertari | 0 | 0 | 0 | 0 | 0 | 0 | 0 |

**Aggression** (number of episodes)

|  | Camila | Tania | Lisala | Limbe | Mirinda | Lolaya | Nefertari |
| --- | --- | --- | --- | --- | --- | --- | --- |
| Camila | 0 | 1 | 2 | 1 | 0 | 7 | 0 |
| Tania | 0 | 0 | 0 | 3 | 0 | 3 | 5 |
| Lisala | 0 | 0 | 0 | 3 | 0 | 2 | 2 |
| Limbe | 0 | 1 | 0 | 0 | 1 | 9 | 3 |
| Mirinda | 0 | 0 | 0 | 0 | 0 | 0 | 0 |
| Lolaya | 0 | 0 | 0 | 0 | 0 | 0 | 0 |
| Nefertari | 0 | 0 | 0 | 0 | 0 | 0 | 0 |
